# Targeting cancer expressed EGFR with a humanized monoclonal antibody

**DOI:** 10.1038/s41598-026-46245-y

**Published:** 2026-03-28

**Authors:** Tamara G. Fernandes Costa, Robert Sarnovsky, Jingyu Zhan, Carolyn A. Maslanka, Ifechukwu Obiorah, Di Xia, David FitzGerald, Antonella Antignani

**Affiliations:** 1 Laboratory of Molecular Biology, Center for Cancer Research, National Cancer Institute, National Institutes of Health, Bethesda, MD 20892 USA; 2 Laboratory of Cell Biology, Center for Cancer Research, National Cancer Institute, National Institutes of Health, Bethesda, MD 20892 USA

**Keywords:** EGFR, Antibody, Tumor, Target, Antibody-antigen complex, Conformation, Biochemistry, Cancer, Drug discovery, Oncology

## Abstract

The humanization of mouse monoclonal antibodies targeting tumor antigens allows for the safe and repeated administration of therapeutic antibodies to humans. Previously, we reported on mouse monoclonal antibody 40H3 and its reactivity with a conformational epitope exposed on EGFR when expressed by cancer cells. To advance 40H3 toward the clinic, we generated various humanized variants, evaluated each for binding to either the peptide epitope or intact cells, and now report on the lead candidate, A10, which exhibited the strongest cell binding activity. A10 binding was characterized in detail on a collection of cancer cell lines each harboring different EGFR mutations or overexpressed EGFR and, where appropriate, compared with 40H3 or Cetuximab. A structural study of the A10 Fab with bound peptide revealed that A10 was unlikely to react with either tethered or untethered forms of wild type EGFR, as the accessibility to the peptide epitope by A10 is EGFR conformational dependent. As a proof of concept to produce therapeutic agents from A10, an antibody drug conjugate (ADC) with monomethyl-auristatin-E (MMAE) was generated and proved potently cytotoxic for cells displaying high levels of EGFR.

**Supplementary Information:**

The online version contains supplementary material available at 10.1038/s41598-026-46245-y.

## Introduction

The epidermal growth factor receptor (EGFR) has been studied intensively as a cancer target for more than four decades^1,2^. EGFR is an oncogenic cell surface protein, driving cancer progression via mutation or overexpression^3^. High affinity ligands for EGFR include EGF, TGFα, while low affinity ligands include epiregulin and epigene^4^. Ligand binding to the receptor’s extracellular domain (ECD) promotes dimerization and activates an intracellular kinase domain leading to phosphorylation of a C-terminal tail rich in tyrosine residues^4–6^.

EGFR gene amplification was discovered in biopsy samples from glioblastoma (GBM) patients^7^. Since then, this genomic alteration has been observed in a subset of epithelial tumors including non-small cell lung cancer (NSCLC), triple negative breast cancer (TNBC)^8^ and colorectal cancer (CRC)^9,10^ resulting in EGFR overexpression. Moreover, in 50% of patient with GBM, EGFR amplification is evident in tumor samples; as is the substantial expression of EGFRvIII, a truncated variant of EGFR^11,12^. EGFRvIII is a truly cancer-specific variant of EGFR where exons 2–7 are deleted and exon 1 is fused with exon 8, which is not seen in normal cells^13^. EGFRvIII which is constitutively active at low level as a kinase, is mostly specific for GBM and is thus a very attractive target for treating this disease.

Because of its role in cancer promotion, EGFR has been the focus of therapeutics that block ligand binding or interfere with kinase-driven signaling^14–17^. An obvious drawback to these therapies is their collateral targeting of wildtype EGFR expressed on normal cells and tissues. An ideal targeting agent might be one with reactivity for both cancer-expressed EGFR and EGFRvIII while exhibiting little or no reactivity for wild type EGFR expressed at normal levels. In this regard, antibodies to EGFR have mainly been developed targeting overexpressed EGFR^18–20^ or EGFRvIII^21^. Previously, we published on the development of 40H3, a mouse monoclonal antibody that reacts with the 287–302 loop of EGFR on cancer cells^20^. This peptide is fully exposed on EGFRvIII and conformationally exposed on wild type EGFR when it is overexpressed on the surface of cancer cells^19^. To develop a humanized version of 40H3 with the goal of improving its utility as a clinical candidate, a total of fourteen humanized candidate antibodies were evaluated and the antibody, designated A10, was the best cell-binding candidate and here we report its characterization.

## Results

### Generation and evaluation of humanized variants of the 40H3 antibody

Bioinformatic modeling together with molecular engineering make it possible to modify monoclonal antibodies of rodent origin and generate humanized antibodies that are less immunogenic (for humans). Due to the nature of the antigen binding site, antibody variable domains, displaying unique complementarity-determining regions (CDRs), will always carry the risk of provoking anti-idiotype antibodies. Regardless, significant safety gains can be made through humanization of the rodent variable (V) domains where the challenge involves maintaining the correct packing of the CDRs, to retain full antigen binding while preserving structural stability.

The strategy to generate recombinant humanized antibody candidates consisted of grafting CDRs onto properly chosen human antibody frameworks. The sequence of the parental antibody, 40H3, was analyzed by homology modelling. Considering the similarity of the parental variable heavy (VH) and variable light (VL) chains with the human germlines IgHV2-26*01 and IgkV1-12*01, mutations were introduced in the mouse variable region generating 3 different humanized variable heavy chains (VH1, VH2, VH3) and 3 different light chains (VL1, VL2, VL3) (Suppl. Figure 1a). The variable heavy and light chain candidates were then fused onto a human IgG1CH/Ig kappa CL acceptor (Suppl. Figure 1b). Overall, a chimeric variant, consisting of the original 40H3 VH and VL and 14 humanized antibodies were generated by pairing 3 heavy chains (VH1, VH2, VH3) and 3 light chains (VL1, VL2, VL3) and two additional variants designed to eliminate potential detrimental posttranslational modifications (PTM) (Table 1). Specifically, in the VL sequence, the parental aspartate at position 56 and the glutamate at position 57 were substituted with an alanine and glycine respectively to avoid the risk of isomerization (Suppl. Figure 1a and Table 1).

**Table 1:** Binding kinetics of antibodies to immobilized EGFRvIII.

Ligand	Antibody	Variable Chain combination	ka (1/Ms)	kd (1/s)	KD (nM)
EGFRvIII	A1	VH + VL	3.38E + 05	8.55E-04	2.53
EGFRvIII	A2	VH1 + VL1	2.09E + 05	9.48E-04	4.53
EGFRvIII	A3	VH1 + VL2	1.89E + 05	1.01E-03	5.35
EGFRvIII	A4	VH1 + VL3	2.85E + 05	9.18E-04	3.22
EGFRvIII	A5	VH2 + VL1	2.01E + 05	8.86E-04	4.4
EGFRvIII	A6	VH2 + VL2	1.86E + 05	7.08E-04	3.81
EGFRvIII	A7	VH2 + VL3	2.20E + 05	8.70E-04	3.95
EGFRvIII	A8	VH3 + VL1	2.06E + 05	6.66E-04	3.24
EGFRvIII	A9	VH3 + VL2	1.90E + 05	7.29E-04	3.84
EGFRvIII	A10	VH3 + VL3	2.44E + 05	6.16E-04	2.53
EGFRvIII	B1	VH + VL-DA	3.74E + 05	7.46E-04	2
EGFRvIII	B2	VH + VL-EG	3.63E + 05	8.92E-04	2.46
EGFRvIII	B3	VH2 + VL2-DA	2.08E + 05	8.27E-04	3.97
EGFRvIII	B4	VH2 + VL1-DA	2.41E + 05	7.16E-04	2.97
EGFRvIII	B5	VH3 + VL1-DA	2.34E + 05	5.95E-04	2.54

**Fig. 1:**
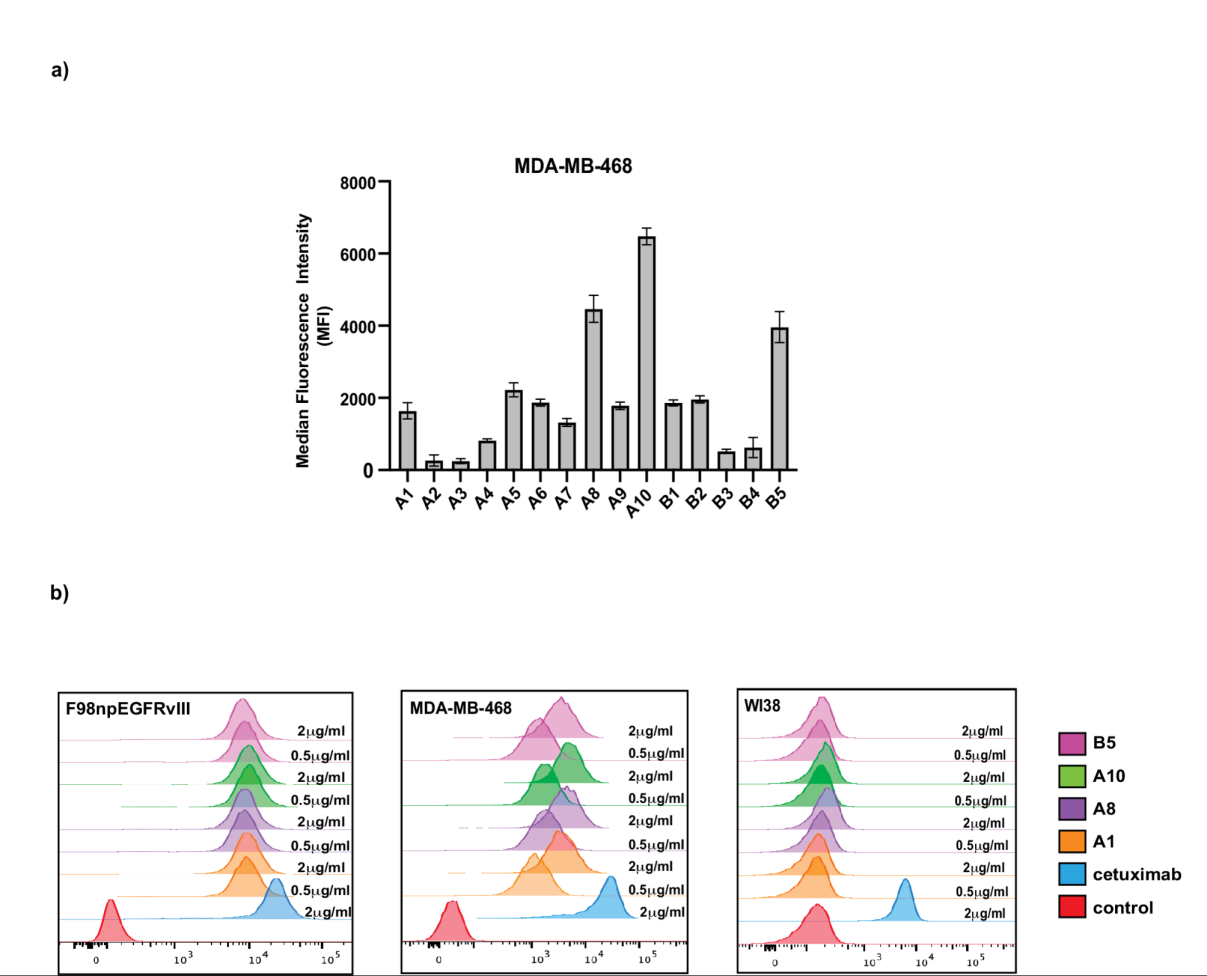
Binding of the humanized antibodies to EGFR. (**a**) Binding of the humanized antibodies at a 2 µg/ml to MDA-MB-468 cells. The bar represented by A1 was the chimera. Data represents the average of three different experiments. *p* values comparing A8, A10 and B8 to the chimera are < 0.001. (**b**) The best EGFR binders were tested on cells stable expressing EGFRvIII (F98npEGFRvIII), wild type EGFR (WI38) and amplified EGFR (MDA-MB-468) at 0.5 and 2 µg/ml. Cetuximab was used at a concentration of 2 µg/ml. The control is represented by the unstained cells. The figure is representative of three experiments.

Once produced, the humanized antibodies showed roughly similar affinity for immobilized EGFRvIII protein (Table 1). The affinity of each candidate was comparable to the chimeric antibody (A1) which contained the mouse variable regions fused to human constant regions (Table 1) and to the original mouse antibody 40H3^20^. To evaluate cellular reactivity, we first tested the binding of each humanized variant to the triple negative breast cancer cell line, MDA-MB-468, which carries a gene amplification for EGFR and overexpresses this receptor on the cell surface (Fig. 1). While all the humanized candidates retained cell binding, three (A8, A10 and B5) exhibited increased binding compared with the chimera. Of these three, A10 was the best binder, followed by A8 and B5 (Fig. 1a). It was notable that all three shared the same variable heavy chain, VH3 (Table 1). Our focus next turned to cells expressing EGFRvIII. On F98 cells stably expressing EGFRvIII, cetuximab showed the highest binding while A10, A8 and B5 followed closely behind, showing near comparable binding (Fig. 1b). When evaluated on MDA-MB-468 cells, A10, A8 and B5 each bound to a lesser extent compared with cetuximab, while demonstrating considerable binding compared to control. In contrast to this, relative to cetuximab, the candidate humanized antibodies showed little or no binding to WI-38 cells which are a normal fibroblast-like line expressing a physiological level of wild type EGFR (Fig. 1b). A possible reason for high binding to EGFRvIII and overexpressed EGFR and low binding to wild type EGFR might relate to the exposure of the target epitope recognized by these humanized variants. Our strategy to humanize the original mouse antibody preserved both the antigen-binding activity and the specificity for cancer expressed EGFR and EGFRvIII. Going forward, we focused on the A10 antibody because of its superior cell-binding property.

### A10 binding to cancer cell lines expressing various forms of EGFR

**Fig. 2:**
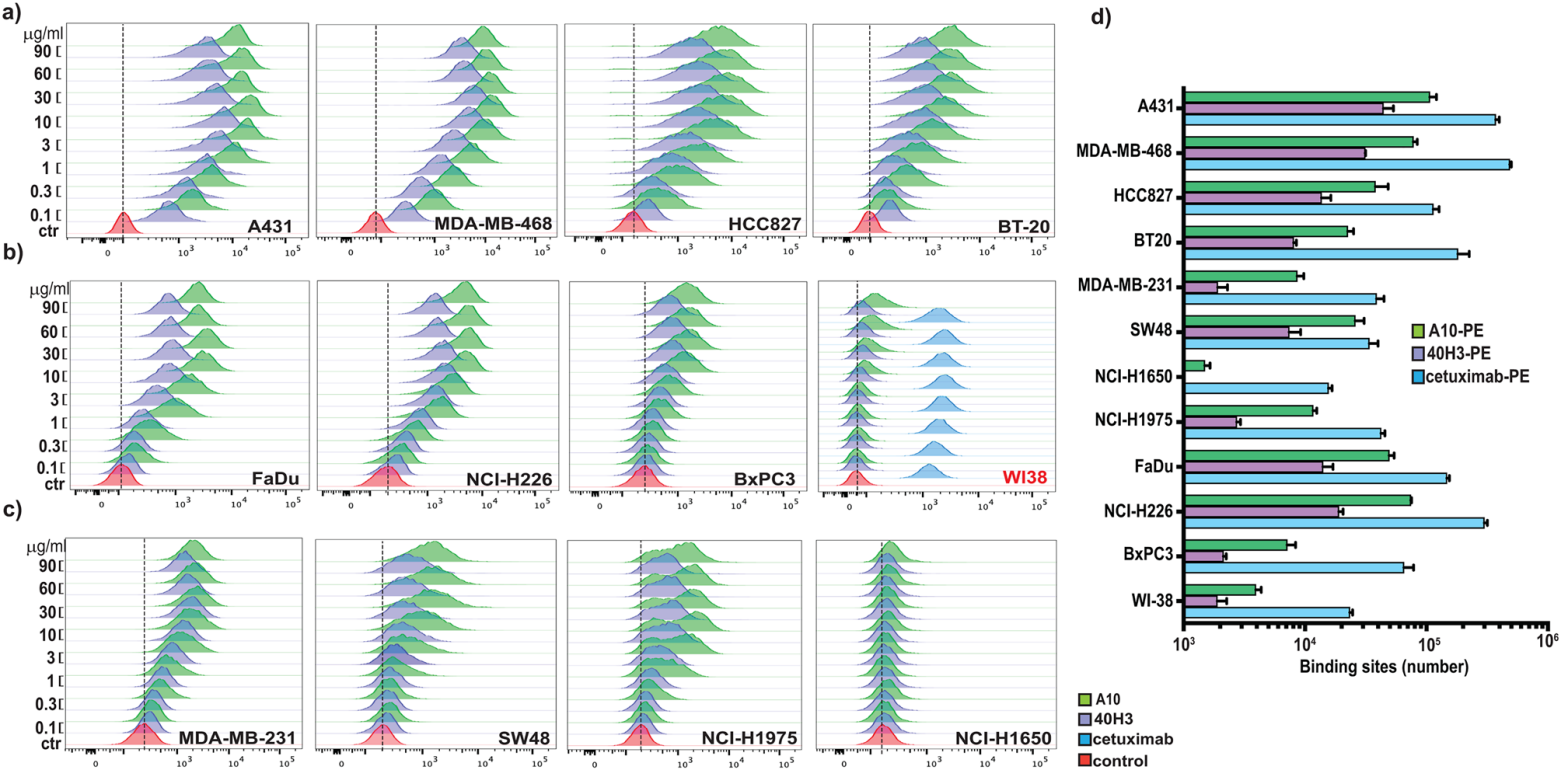
A10 binding to EGFR. The binding affinity of A10 or 40H3 was determined on amplified (**a**), wild type overexpressed (**b**) and mutated EGFR-expressing tumor cells (**c**) by flow cytometry analysis. Cetuximab was used as positive control for wild type EGFR expressed on the normal fibroblast cell line, WI38. The antibodies were tested in a dose dependent manner as indicated (0.1–90 µg/ml). (**d**) The number of binding sites per cell was determined using each PE-labeled antibody at a concentration of 10 µg/ml.

To expand our initial evaluation, we undertook a broader and more detailed analysis of the A10 reactivity on human tumor cell lines carrying various EGFR mutations. We chose cell lines with EGFR gene amplification (MDA-MB-468, A431, BT20 and HCC827), point mutations (MDA-MB-231, NCI-H1650, SW48 and NCI-H1975) and cell lines with wild type but overexpressed EGFR. These cell lines were derived originally from various human epithelial cancers including breast, colorectal, head and neck, pancreas and lung cancers. As a negative control we included the non-cancerous human fibroblast cell line WI38 (Table 2). Not surprisingly, cell lines with amplified EGFR had a greater expression of the receptor based on both RNA and protein levels compared to the other lines. It is notable that, while the EGFR copy number was responsible for a substantial protein level, there was not a clear correlation between reported copy number and expression level as A431 cells showed an EGFR gene copy number similar to BT20 but EGFR expression level was considerably higher in A431 cells (Table 2). Expression levels generally appeared to influence the binding of A10 and 40H3 for EGFR (Table 2; Fig. 2). Gene-amplified EGFR cell lines, such as MDA-MB-468, A431, and HCC827, showed higher binding capacity compared to the other cell lines (Fig. 2a). Lower binding was associated with a reduced level of receptor expression. In this regard, BT20 exhibited a similar binding pattern compared to wild type EGFR expressed on FaDu and NCI-226 cells (Fig. 2b). The affinity for both A10 and 40H3 was minimal for MDA-MB-231, NCI-H1650, SW48 and NCI-H1975 (Fig. 2c) cells. Of note, the non-cancerous cell line, WI38, expressed EGFR, as revealed by the substantial binding of cetuximab, but didn’t bind A10 (Fig. 2b), confirming that A10 is specific for EGFR expressed on malignant cells. It was also evident that A10 was a better binder than 40H3, attributable possibly to the role of human framework residues introduced during the humanization process.

**Table 2:** Binding site number for A10, 40H3 and Cetuximab on tumor cell lines. EGFR expression (TPM), EGFR protein level and known mutations, altered gene copy number and single nucleotide polymorphism (SNP) are from Cell Passport (https://cellmodelpassports.sanger.ac.uk/). NA: not applicable.

	TPM	Protein Intensity	EGFR Mutation	Copy number	A10 (binding site)	40H3 (binding site)	Cetuximab (binding site)
A431	9.80	9.53	amplification	16.61	106,428	44,137	370,765
MDA-MB-468	9.18	8.89	amplification	73.09	77,839	31,216	484,608
HCC827	9.67	8.02	amplification	34.04	37,859	13,668	113,628
BT20	7.82	7.16	amplification	12.79	22,476	8,040	181,325
SW48	6.90	5.53	SNP	NA	25,845	7,395	33,806
NCI-H1975	5.32	4.18	SNP	NA	11,656	2,726	42,218
MDA-MB-231	5.73	5.30	SNP	NA	8,602	1,910	38,755
NCI-H1650	6.08	5.76	Deletion	NA	1,495	192	15,583
NCI-H226	6.36	6.15	wild type	NA	74,226	18,976	299,442
FaDu	6.55	6.28	wild type	NA	49,179	14,034	146,998
BxPC3	5.97	7.33	wild type	NA	7,114	2,124	65,130
WI-38	na	na	wildtype	NA	3,927	1,892	23,346

We quantified the number of binding sites for the A10 antibody and compared them to 40H3 and cetuximab (Fig. 2d; Table 2). From high to low, A431 cells had 106,428 binding sites for A10 followed by MDA-MB468 and NCI-H226 with ~ 70,000 sites per cell. FaDu cells had almost 50,000 sites, followed by HCC827 cells with 37,000 sites. BT20 and SW48 exhibited ~ 20,000 and NCI-H1975 11,000 sites/cell. All the other cell lines had less than 10,000 sites/cell. In particular, the NSCLC line, NCI-H1650, had few sites and was in the same range as WI38 cells. Universally, for all cell lines tested, the binding site number per cell for A10 was higher than for 40H3 from the highest number of sites/cells (such as A431cells) to the smallest number (for NCI-H1650 and WI38 cells). This result could be likely attributed to the 40H3 lower affinity for EGFR (Fig. 2a-c). When making changes to antibody sequence, the concern is always loss of function. Here the humanization changes produced an antibody with improved binding over the original 40H3. The fold increase for each cell line varied from 3 to 10 fold with no correlation to EGFR mutation status. Generally, for cell lines, except for SW48, cetuximab exhibited more binding sites per cell than A10.

Considering that, in the extracellular domain, cetuximab blocks ligand binding while the A10 antibody targets the loop of aa 287–302 which is fully exposed in EGFRvIII (hence the near parity of binding with cetuximab on F98-EGFRvIII cells) or on amplified EGFR predominantly on tumor cells^22,23^, we speculate that cetuximab can bind EGFR without exception due to the location of its target while A10 specifically recognizes a subset of receptors where the target loop is exposed preferentially on tumor cells.

### Structure of the Fab(A10) in complex with EGFR peptide

**Fig. 3:**
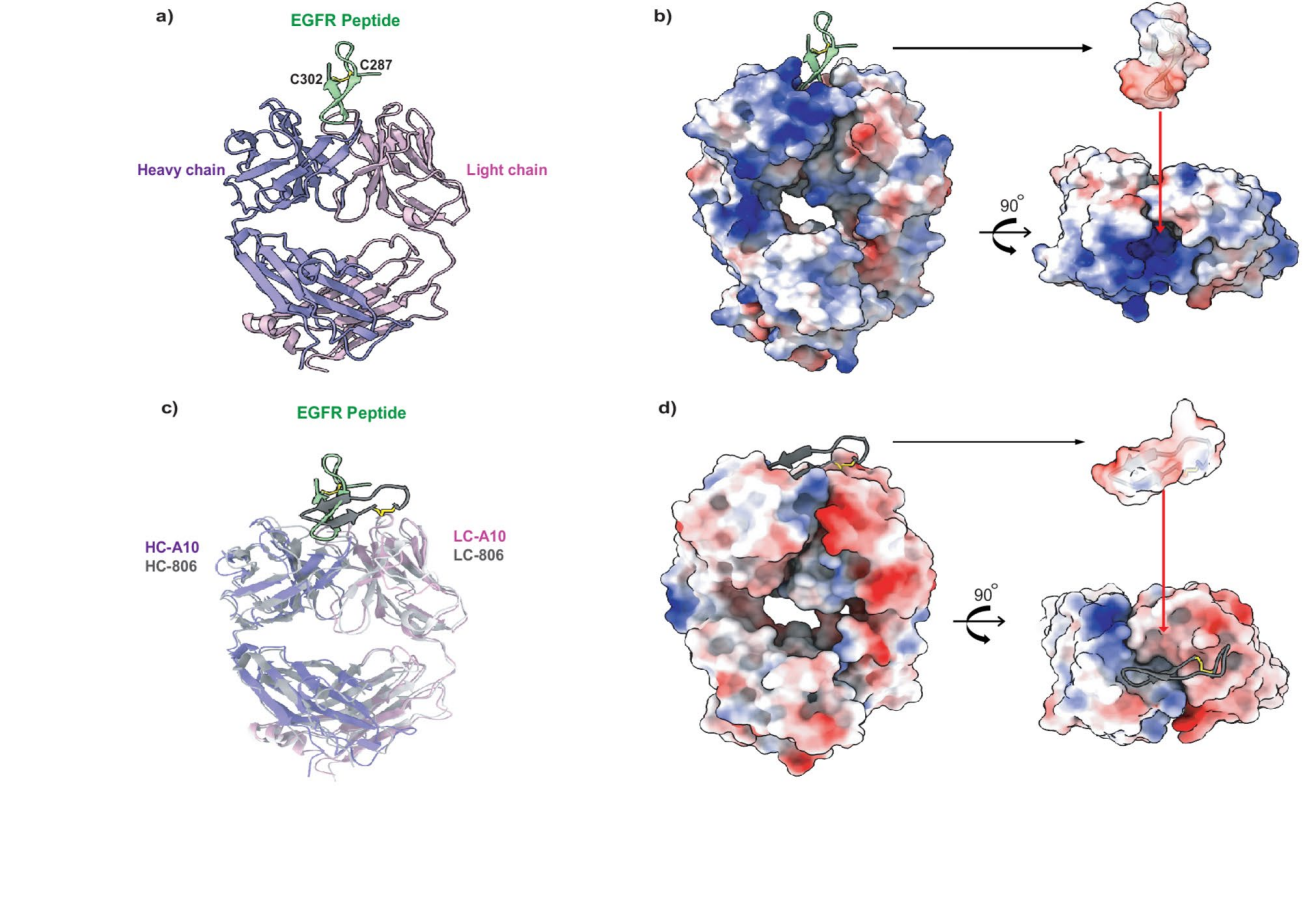
Structure of Fab(A10)/EGFR-derived peptide. (**a**) Structure of Fab(A10)/peptide in ribbon representation. The heavy chain is in purple and the light chain in pink. The bound peptide is in a β-hairpin conformation and colored green. The disulfide bond formed between residues C302 and C287 is shown in yellow sticks and labeled. (**b**) Surface representation of Fab(A10). The structure of Fab(A10)/peptide is shown in the same orientation as in (a), with the antibody rendered as molecular surface, which is colored by electrostatic surface potential. A red surface is a negative potential, whereas a blue surface is a positive potential. White surfaces are neutral. The electrostatic potential surface of the bound peptide is represented in the same orientation as in (a) or rotated 90°, showing the charge complementarity of antibody binding surface with that of the peptide epitope. (**c**) Superposition of A10/peptide with 806/peptide. The light chain (LC) and heavy chain (HC) of A10 are colored purple and blue, respectively. The LC and HC of 806 are in light and dark gray. The peptide bound to A10 is colored green and that of 806 in black. d) Electrostatic potential surface of Fab(806) in the same orientation as in (c). The bound peptide is rendered as a cartoon given in two orthogonal orientations. The electrostatic potential surface of the bound peptide in the same orientation as in (c) and rotated 90° showing the charge complementarity of antibody binding surface with that of the peptide epitope.

To investigate the atomic features of A10 binding to the original immunizing EGFR peptide, we generated a Fab fragment and co-crystallized it with the oxidized disulfide loop peptide (residues 287–302), which was flanked with A286 and K303 to bookend the cystines with their native amino acid neighbors. The structure was determined to 2.45 Å resolution, revealing two nearly identical Fab(A10)/EGFR peptide complexes (rms deviation = 0.68 Å when superposed) in the crystallographic asymmetric unit. All 18 residues of the EGFR-derived peptide were clearly traceable in the electron density map, adopting a β-hairpin conformation that was aided by the disulfide bond formed between C287 and C302 (Fig. 3a).

Within the EGFR peptide, residues A286-Y292 were solvent-exposed with relatively weaker electron densities and were not involved in antibody interaction, while residues E293-C302 formed a well-defined anti-parallel β-hairpin that was wedged into the groove between the variable regions of the Fab heavy and light chains (Fig. 3a), constituting the epitope for antibody binding. The strong association of the peptide with the Fab(A10) is corroborated with a total buried solvent-exposed surface area of 1,129.4 Å^2^, for which the peptide contributed 604.2 Å^2^, and the heavy and light chain contributed 290.8 Å^2^ and 234.4 Å^2^, respectively (Table 3). Major interactions between the peptide and the antibody were mediated by H-bonding and charged interactions, as the folded peptide displayed a charge dipole moment with negatively charged side chains interacting with the antibody (Figure. 3b, Suppl. Figure 2, and Table 3). Complementing the negative potential tip of the peptide was the positive surface potential cavity on the binding surface of the antibody (Fig. 3b). Specifically, the negatively charged E295 and E296 side chains of the EGFR peptide formed salt bridges with K98 in the CDR3 and R53 in the CDR2 of the heavy chain, respectively, in addition to hydrogen bonds and electrostatic interactions formed between the EGFR peptide and A10 Fab (Table 3 and Suppl. Figure 2), most of which involved charged residues.

**Table 3:** Interactions between the EGFR peptide and A10 Fab. Where indicated (*) are the main chain atoms. CDRs represent the Complementarity Determining Regions. Van der Waals’ contacts are not listed.

EGFR peptide residue	Light Chain residue	Heavy Chain residue	CDRs	Distance (Å)	Interactions
E293 (OE1)	W32 (NE1)		L1	3.04	H-bond
E295 (OE1,OE2)		K98 (NZ)	H3	3.09, 3.37	Ionic interaction
D297		G33 (N*)	H1	2.97	H-bond
D297		R53 (N*)	H2	3.11	H-bond
D297(O*)		H35 (NE2)	H1	2.71	H-bond
G298 (O*)		G103 (N*)	H3	3.13	H-bond
R300 (N*)	L91(O*)		L3	3.03	H-bond
R300 (NH1)	Y92(O*)		L3	2.7	H-bond
R300 (NH1)	Y92 (CE2)		L3	3.67	Cation-π interaction
K301 (NZ)		W52 (CD2)	H2	3.38	Cation-π interaction
604.2	234.4	290.8			Buried surface area (Å^2^)

The 16-residue epitope (C287-C302) recognized by A10 has previously been targeted by the monoclonal antibody, 806^24^. A structural superposition of A10/peptide with 806/peptide (PDB: 3G5V) gave rise to rms deviations of 1.41 Å and 1.65 Å, respectively, for the light and heavy chains. While the antibodies A10 and 806 aligned well, the two bound peptides in similar β-hairpin conformations, were completely misaligned (Fig. 3c), reflecting distinct binding modes due to different ways the peptide interacted with each antibody. To bind A10, the peptide took a diving pose with the negatively charged side penetrating deep into the antibody. There was a corresponding surface charge complementarity between the peptide and the A10 binding surface (Fig. 3b). On the other hand, the peptide laid flat on the binding surface of 806 with predominantly one side of the β-hairpin in contact with the antibody. Accordingly, there was also a surface charge complementarity between the peptide and the 806 binding surface (Fig. 3d).

When interacting peptide residues were surveyed and compared, we noted that the peptide β-hairpin used a combination of distinct and similar contact residues to interact with either A10 or 806 (Suppl. Table 1). Counting only H-bonds and charged interactions, we noticed that three peptide residues E295, E296, and G298 engaged exclusively with A10, whereas two different peptide residues A289 and C302 interacted with 806. In contrast, the following peptide residues E293, D297, R300, and K301 were shared by A10 and 806. We also noted that peptide residues (293–301), interacting with A10, were clustered at one end of the peptide β-hairpin, while those interacting with 806 contacted both ends of the β-hairpin (Fig. 3c-d, Suppl. Table 1). We conclude that the differences in the amino acid sequences of the CDRs between A10 and 806 led the antibodies to interact in two distinct ways with the same EGFR peptide β-hairpin.

### Implications for the interactions between A10 and EGFR

EGFR extracellular domain (ECD) consists of four structural subdomains: domains 1 and III are involved in ligand binding and domains II and IV are cystine rich domains forming a series of disulfide bridges^1^. In the absence of a ligand (like EGF or TGFα), EGFR exists predominantly in a tethered conformation^25,26^. In this conformation, the ectodomain of EGFR folds with an intramolecular “tether” formed by the interaction of domains II and IV. This tether essentially autoinhibits the receptor, preventing it from forming dimers and activating its kinase activity^27^. The presence of a ligand triggers conformational changes that disrupts the intramolecular tether, exposing the dimerization arm located in domain II, allowing the receptor to adopt an untethered conformation leading to dimerization^28,29^, (Suppl. Movie 1) followed by autophosphorylation of the intracellular tyrosine kinase domains, initiating downstream signaling cascades.

To address the question of whether the EGFR peptide bound to A10 has the same β-hairpin structure as in full-length EGFR, we performed pairwise superposition of the A10-peptide with EGFR-derived structures^25,26,28,29^. The comparisons gave rise to rms deviations in the range of 0.7 to 1.7 Å (Suppl. Tables 2 and Suppl. Figure 3). Further, it should be noted that in the structures reported for EGFRvIII, the 287–302 peptide was not resolved, suggesting its flexibility in this truncation variant^30^. The small rms deviation values indicate that the β-hairpin structure of the oxidized peptide complexed with A10 is basically similar to the one in the full-length ectodomain of EGFR, suggesting that A10 binding did not distort the peptide structurally.

**Fig. 4:**
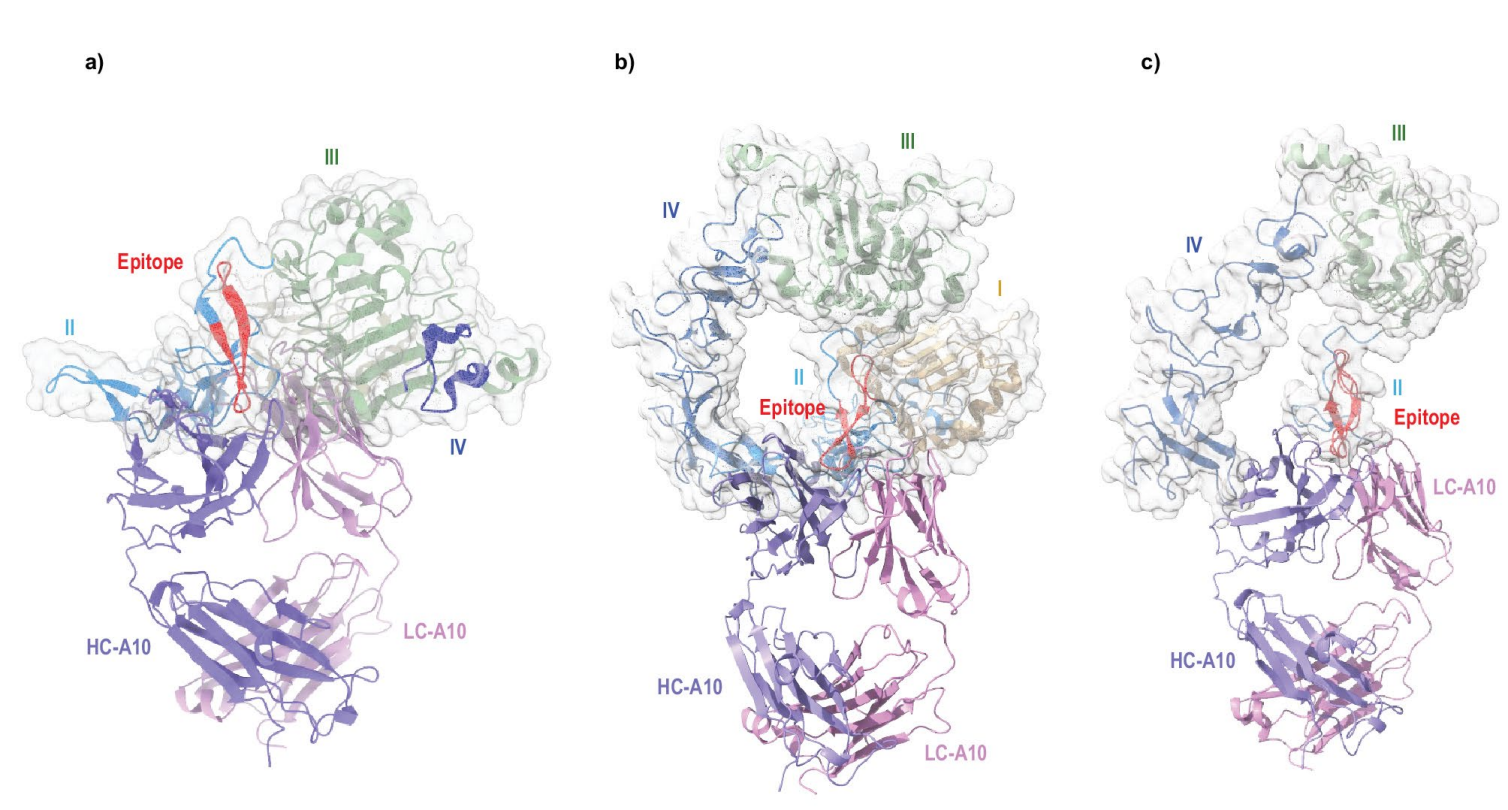
Molecular modeling of A10 binding to the ectodomain of EGFR. (**a**) Modeled binding of A10 to a single ectodomain of EGFR in an untethered state derived from 1MOX. The modeling of A10 binding is based on structural alignment of the common peptide epitope. The four domains of EGFR are shown as cartoon in different colors: domain I (1–164, wheat), Subdomain II (165–310, cyan), domain III (311–480, green), and domain IV (481–618, blue). The EGFR ectodomain is also drawn as a semitransparent van der Waals surface in gray overlaying the cartoon diagram. The heavy chain is colored purple while the light chain is pink. (**b**) Modeling of A10 binding to a single ectodomain of EGFR in a tethered state derived from 1NQL. (**c**) Modeling of A10 binding to EGFRvIII. By removing first 273 residues from the monomeric ectodomain of EGFR (PDB:1NQL), a model for EGFRvIII can be generated, containing part of Domain II (cyan), Domain III (green), and Domain IV (blue). Alignment of Domain II with the peptide in the Fab/peptide structure gives rise to the model as shown. Data availability. The atomic coordinates have been deposited in the Protein Data Bank (www.pdb.org) under accession code 9Z2H [10.2210/pdb9z2h/pdb].

Having established that A10 does not distort the peptide, we next addressed the issue of antibody access to its epitope. Among available structures of the ectodomain of EGFR, the dimeric, untethered, ligand-bound active receptors represented by PDB:1MOX is conformationally very different from the monomeric, tethered, and inactive one in 1NQL. Structures of these two classes do not superpose with each other. We first superposed the β-hairpin of our A10/peptide structure to the same region of the ectodomain of the monomeric EGFR derived from the dimeric EGFR structure^29^ that is in the presumed untethered state. As modeled, it is not possible for A10 to access the epitope without colliding badly with the rest of the EGFR extracellular domains. Because most collisions are from the same subunit (Fig. 4a), we conclude that the monoclonal antibody A10 will not bind the dimer. To determine if the reactive epitope is accessible to A10 when EGFR is in the monomeric, tethered conformation, we aligned the β-hairpin of A10/peptide structure onto its counterpart in the structure of tethered EGFR (PDB:1NQL^25^. In the tethered conformation, the A10 interacts with EGFR with a much reduced, but not eliminated, number of collisions (Fig. 4b). Thus, neither structure allows for unincumbered docking, leading to the conclusion that the binding epitope of EGFR could be accessible to A10 binding when the EGFR molecule is in a transition conformation from the tethered to the untethered conformations, during which the epitope becomes fully exposed (Suppl. Movie 2).

The reactive epitope is clearly accessible to A10 binding in EGFRvIII. Although structures of EGFRvIII in various forms have recently been reported^30^, the deposited models (for example PDB:8UKX) do not contain the A10 binding epitope, suggesting this part of the EGFRvIII is highly flexible after removing the N-terminal 267 residues. Since EGFRvIII structures only align with those of tethered EGFR ectodomains, we modeled the A10/EGFRvIII interaction using the PDB:1NQL^25^ after truncating its N-terminal 273 residues. The EGFRvIII variant embodies a N-terminal truncation from residues 6–273, eliminating completely domain I and partially domain II, leading to a largely solvent exposed remainder of domain II (cyan in Fig. 4c), which contains the peptide epitope accessible to A10. This result confirmed that A10 is an effective antibody targeting EGFRvIII.

### Producing A10-based therapeutics

A10 was chemically conjugated to the tubulin-inhibitor payload monomethyl auristatin-E (MMAE) through a Val-Cit cleavable linker which contains a valine-citrulline dipeptide cleavable by Cathepsin B. The ADC had a drug-antibody ratio of 3 and the antibody retained full cell binding activity after conjugation (Suppl. Figure 4a).

When we tested on EGFR- high expressing MDA-MD-468 and A431 cells, A10-MMAE was able to induce cell death, confirming that the conjugation did not impair the A10 ability to bind cells. Moreover, the lack of effect observed with the isotype ADC IgG1-MMAE in both cell lines, demonstrated the specificity of our ADC for EGFR (Suppl. Figure 4b).

We evaluated A10-MMAE across cell lines exhibiting differential EGFR expression. The A10-MMAE was most active on cell lines carrying amplified EGFR (Suppl. Figure 4c). As expected by the number of binding sites, A431 cells were killed completely by A10-MMAE (IC50 = 0.62nM). MDA-MB-468 followed with an IC50 of 1.57nM), then HCC827 and BT20 with IC50s in the order of 40nM (Table 4). It’s notable that HCC827 and BT20 have 2–3-fold fewer binding sites than MDA-MB-468 but their IC50s were 30–40 times higher because they were substantially less sensitive to MMAE (Table 4).

**Table 4:** IC50 values for A10-MMAE and MMAE.

IC50	A10-MMAE (nM)	MMAE (nM)
A431	0.62	0.20
MDA-MB-468	1.57	0.29
HCC827	32.05	20.41
BT20	45.93	18.77
SW48	4.43	0.08
NCI-H1975	19.67	3.09
MDA-MB-231	> 100	2.55
NCI-H1650	> 100	> 100
NCI-H226	> 100	16.14
FaDu	> 100	22.45
BxPC3	23.98	1.90
*WI38*	> 100	4.40

In tumor cells with mutated EGFR, the toxicity of the payload was offset by the low number of binding sites. SW48 cells were eliminated by low concentrations of MMAE (IC50 0.04nM) and the result was an IC50 for A10-MMAE similar to MDA-MB-468, despite having only a third of the A10 sites. Moreover, sites for A10 were less abundant in NCI-H1975 than in HCC827 or BT20 (Table 2) but MMAE was more toxic and, as a consequence, A10-MMAE was more potent (IC50 19.67nM). Although they were killed by MMAE, MDA-MB-231 were not eradicated by A10-MMAE possible due to low number of binding sites. Finally, NCI-H1650 cells were resistant to the payload and the ADC and they had the lowest number of A10 sites. Overall, cells expressing wild type EGFR cells were not very sensitive to either the payload or the antibody-drug conjugate, except for BxPC3. These cells were one of the most sensitive to the payload MMAE. Moreover, BxPC3 are wild type Ras cells which mediates signaling downstream of EGFR kinase^31^. Blockade of activated EGFR with clinically used antibodies suppressed the EGFR/wild-type RAS signaling axis^31,32^ and it could contribute also to the ADC cytotoxic effect. Non-cancerous wild type EGFR WI38 fibroblasts were completely resistant to the ADC even though MMAE was very potent. In conclusion, the cytotoxic activity of A10-MMAE is the result of a fine balance between the number of the antibody binding sites and the intrinsic sensitivity to MMAE.

## Discussion

The anti-EGFR antibody 40H3, originally raised in mice, recognizes an epitope contained within a cysteine loop (amino acids 287–302) that is completely accessible in EGFRvIII and exposed on cells overexpressing EGFR and this feature restricted its binding solely to tumor cells^20,33^. Humanization of this antibody was a mandatory step in its development as a cancer therapeutic.

The best candidate antibody from our screening of humanized variants, A10, retained all the properties of the mouse antibody. The introduced mutations converting to a human framework didn’t harm the specificity for EGFR expressed on cancer cells: A10 binds the truncated receptor EGFRvIII and over-expressed wild type EGFR but not wild type EGFR when expressed at low to modest levels on cells.

From the experimental structural resolution of the A10 Fab with the bound EGFR loop (aa287–302), we demonstrated that A10 recognized the same epitope sequence in wild type EGFR, which is also conserved structurally in EGFRvIII. Structural comparison between A10 and 806, both targeting the same loop sequence, revealed striking difference in their selections of binding epitopes. However, the structural environment of the epitope in both monomeric and dimeric EGFR structures would block antibody access. Moreover, considering that in EGFR expressing cells, A10 bound only a fraction of the total EGFR population, we speculate that during ligand activation, the receptor undergoes a substantial domain rearrangement, transitioning from monomeric untethered to dimeric tethered form, exposing the epitope in the process, and adopting a conformation that permits antibody binding (Suppl. Movie 1 and 2). This explains the insignificant binding of A10 to normal cells and the substantially higher levels of binding to tumor cells with overexpressed EGFR.

This study supports the idea of generating antibodies to overexpressed or misfolded receptors exclusively expressed on cancer cells^19^ and as proof of concept the A10-MMAE was toxic to cancer cells expressing high EGFR. Together these data support the development of the A10-based cancer therapeutics.

## Methods

### Antibody humanization design and production

The generation and production of humanized antibodies was carried out at GenScript. The sequence of the parental antibody, 40H3, was modelled with a homology-modelling program and humanized antibodies were designed using CDR grafting. Briefly, the 40H3 CDRs were grafted into the human IgG germline sequences and joined with acceptor constant regions (Supplemental Fig. 1) to obtain candidate humanized light and heavy chains. In total, 3 heavy chain variants (VH1, VH2, VH3) and 3 light chain variants (VL1, VL2, VL3) were paired with each other for affinity ranking experiments. Further, according to GenScriptProbio’s standard operation procedure (SOP), the post transcriptional modification (PTM risk of all sequences was analyzed, and appropriate removal mutations were generated. The DNA sequences encoding antibody heavy and light chains were synthesized and inserted into the pcDNA3.4 vector to construct expression plasmids to produce full-length IgGs. The designed plasmids of heavy chain and light chain were sent for 4 mL transfection following Probio’s SOP. Expression of antibody was produced via CHO cell culture and the antibody-containing supernatants were purified using a protein A affinity column. The purified antibodies were buffer exchanged into PBS using a Zeba desalting column. The concentration and purity of each antibody were determined by OD280 and SDS-PAGE, respectively. The affinity of purified antibody binding to antigen was individually determined using a Surface Plasmon Resonance (SPR) biosensor, Biacore 8 K (Cytiva). The antigen was immobilized on the sensor chip and antibodies were used as analytes. Dissociation (kd) and association (ka) rate constants were obtained using Biacore 8 K evaluation software (Biacore™ Insight Evaluation Software). The equilibrium dissociation constants (KD) were calculated from the ratio of k*d* over k*a* (Table 1).

### Cell lines

All the cell lines were purchased from ATCC. A431, MDA-MB-468, SW48, MDA-MB-231 and F98npEGFRvIII were cultured in DMEM (ThermoFisher Scientific, NY, USA). HCC827, NCI-H1975, NCI-H1650, NCI-H226 and BxPC3 were cultured in RPMI media (ThermoFisher Scientific, NY, USA). BT20, FaDu and WI38 were cultured in EMEM (ATCC, VA, USA). All the media were supplemented with 10% fetal bovine serum (ThermoFisher Scientific, NY, USA), 2 mM GlutaMAX (ThermoFisher Scientific, NY, USA), MEM non-essential amino acids (NEAA) (ThermoFisher Scientific, NY, USA). The medium for F98npEGFRvIII cells was further supplemented with 0.2 mg/ml G-418 (ThermoFisher Scientific, NY, USA). Cells were grown at 37 ◦C with 5% CO2. Transcripts Per Million (TPM) are from Cell Passport website (https://cellmodelpassports.sanger.ac.uk). RNA-seq data were collated from the Wellcome Sanger Institute and the Broad Institute (https://depmap.org/portal/data_page/?tab=allData). Read counts and TPM (transcripts per million) values for both data sources were inferred from using the RSEM tool (10.1186/1471-2105-12-323). TPM values are reported after log2 transformation, using a pseudo-count of 1; log2(TPM + 1). Data presented through the Cell Model Passports website combines the Sanger and Broad datasets. Where cell models have been screened at both institutes, the Sanger data has been selected for the merged dataset. Protein intensity is from Cell Passport from a “in silico” spectral library created using DIA-NN (version 1.8) (Demichev et al. 2020) for the canonical human proteome (Uniprot Release 2021_03; 20,612 sequences), along with retention time peptides and commonly occurring microbial and viral sequences. The final dataset, termed ProCan-DepMapSanger, was derived from 6,864 mass spectrometry runs covering 949 cell lines and quantifying a total of 8,498 proteins.”

### Preparation of Fab-peptide complex

The fab fragment of the A10 antibody was prepared using the Pierce Fab preparation kit (Thermo Fisher Scientific) following the manufacture’s instruction as previously described (Zhan et al., 2023, CRC). Briefly, monoclonal antibody A10 was digested by resin-immobilized papain at 37 °C for 4 h. Cleaved Fab fragment was separated from Fc fragment and undigested IgG using a Protein A column. Flow-through from the Protein A column which contains purified Fab was pooled and concentrated to 10.23 mg/ml using an Amicon Ultra concentrator (Millipore) with a molecular weight cutoff of 30 kDa. The lyophilized EGFR peptide (sequence ACGADSYEMEEDGVRKCK, residues 286–303 of EGFRvIII (GenScript) was dissolved in buffer containing 20 mM Tris, 100 mM NaCl, pH7.5 to a final concentration of 10 mg/ml. The EGFR peptide solution was mixed with concentrated Fab at a molar ratio of 4:1 and left on a horizontal orbital shaker at 4 °C overnight before setting up the crystallization trays.

### Crystallization of Fab-peptide complex

The crystallization was performed by the hanging-drop method robotically. An aliquot of 0.24 µl protein complex was mixed with equal volume of reservoir solution (100 µl) containing 0.8 M NaH_2_PO_4_, 1.2 M KH_2_PO_4_, and 0.1 M Na Acetate, pH 4.5. Three-dimensional crystals appeared 50 days after incubating at 4 °C. Crystals were flash frozen in liquid nitrogen prior to data collection.

### X-ray diffraction data collection, structure determination and refinement

X-ray diffraction dataset was collected at 100 K using beamline at the National Synchrotron Light Source II (NSLS-II), Brookhaven National Laboratory (BNL). Diffraction data images were processed using HKL2000^34^. The structure was solved by the molecular replacement (MR) method using the BALBES program^35^ in the CCP4 program suite^36^. Atomic model was built using the program COOT^37^ and refined using Phenix^38^.

## Supplementary Information

Below is the link to the electronic supplementary material.

Supplementary Material 1

Supplementary Material 2

Supplementary Material 3

## Data Availability

The atomic coordinates have been deposited in the Protein Data Bank (www.pdb.org) under accession code 9Z2H [https://doi.org/10.2210/pdb9z2h/pdb].

## References

[1] Lemmon, M. A., Schlessinger, J. & Ferguson, K. M. The EGFR family: Not so prototypical receptor tyrosine kinases. *Cold Spring Harb. Perspect. Biol.* **6**(4), a020768. 10.1101/cshperspect.a020768 (2014). 24691965 10.1101/cshperspect.a020768 PMC3970421

[2] Schultz, D. F., Billadeau, D. D. & Jois, S. D. EGFR trafficking: Effect of dimerization, dynamics, and mutation. *Front. Oncol.* **13**, 1258371. 10.3389/fonc.2023.1258371 (2023). 37752992 10.3389/fonc.2023.1258371 PMC10518470

[3] Pines, G., Kostler, W. J. & Yarden, Y. Oncogenic mutant forms of EGFR: Lessons in signal transduction and targets for cancer therapy. *FEBS Lett.* **584**(12), 2699–2706. 10.1016/j.febslet.2010.04.019 (2010). 20388509 10.1016/j.febslet.2010.04.019 PMC2892754

[4] Freed, D. M. et al. EGFR Ligands Differentially Stabilize Receptor Dimers to Specify Signaling Kinetics. *Cell* **171**(3), 683-695 e18. 10.1016/j.cell.2017.09.017 (2017). 28988771 10.1016/j.cell.2017.09.017 PMC5650921

[5] Du, Z. & Lovly, C. M. Mechanisms of receptor tyrosine kinase activation in cancer. *Mol. Cancer* **17**(1), 58. 10.1186/s12943-018-0782-4 (2018). 29455648 10.1186/s12943-018-0782-4 PMC5817791

[6] Brown, B. P. et al. Allele-specific activation, enzyme kinetics, and inhibitor sensitivities of EGFR exon 19 deletion mutations in lung cancer. *Proc. Natl. Acad. Sci. U. S. A.* **119**(30), e2206588119. 10.1073/pnas.2206588119 (2022). 35867821 10.1073/pnas.2206588119 PMC9335329

[7] Libermann, T. A. et al. Amplification and overexpression of the EGF receptor gene in primary human glioblastomas. *J. Cell Sci. Suppl.* **3**, 161–172. 10.1242/jcs.1985.supplement_3.16 (1985). 3011820 10.1242/jcs.1985.supplement_3.16

[8] Park, H. S. et al. High EGFR gene copy number predicts poor outcome in triple-negative breast cancer. *Mod. Pathol.* **27**(9), 1212–1222. 10.1038/modpathol.2013.251 (2014). 24406864 10.1038/modpathol.2013.251

[9] Takano, T. et al. Epidermal growth factor receptor gene mutations and increased copy numbers predict gefitinib sensitivity in patients with recurrent non-small-cell lung cancer. *J. Clin. Oncol.* **23**(28), 6829–6837. 10.1200/JCO.2005.01.0793 (2005). 15998907 10.1200/JCO.2005.01.0793

[10] Jiang, Z. et al. EGFR gene copy number as a prognostic marker in colorectal cancer patients treated with cetuximab or panitumumab: A systematic review and meta analysis. *PLoS One* **8**(2), e56205. 10.1371/journal.pone.0056205 (2013). 23441167 10.1371/journal.pone.0056205 PMC3575344

[11] Gan, H. K., Cvrljevic, A. N. & Johns, T. G. The epidermal growth factor receptor variant III (EGFRvIII): Where wild things are altered. *FEBS J.* **280**(21), 5350–5370. 10.1111/febs.12393 (2013). 23777544 10.1111/febs.12393

[12] Felsberg, J. et al. Epidermal growth factor receptor variant III (EGFRvIII) positivity in EGFR-amplified glioblastomas: Prognostic role and comparison between primary and recurrent tumors. *Clin. Cancer Res.* **23**(22), 6846–6855. 10.1158/1078-0432.CCR-17-0890 (2017). 28855349 10.1158/1078-0432.CCR-17-0890

[13] An, Z. et al. Epidermal growth factor receptor and EGFRvIII in glioblastoma: Signaling pathways and targeted therapies. *Oncogene* **37**(12), 1561–1575. 10.1038/s41388-017-0045-7 (2018). 29321659 10.1038/s41388-017-0045-7 PMC5860944

[14] Grunwald, V. & Hidalgo, M. Developing inhibitors of the epidermal growth factor receptor for cancer treatment. *J. Natl. Cancer Inst.* **95** (12), 851–867. 10.1093/jnci/95.12.851 (2003). 12813169 10.1093/jnci/95.12.851

[15] Harari, P. M., Allen, G. W. & Bonner, J. A. Biology of interactions: Antiepidermal growth factor receptor agents. *J. Clin. Oncol.* **25**(26), 4057–4065. 10.1200/JCO.2007.11.8984 (2007). 17827454 10.1200/JCO.2007.11.8984

[16] Capdevila, J. et al. Anti-epidermal growth factor receptor monoclonal antibodies in cancer treatment. *Cancer Treat Rev* **35**(4), 354–63. 10.1016/j.ctrv.2009.02.001 (2009). 19269105 10.1016/j.ctrv.2009.02.001

[17] Vaquero, J. et al. Genetic alterations shaping tumor response to anti-EGFR therapies. *Drug Resist. Updat.* **64**, 100863. 10.1016/j.drup.2022.100863 (2022). 36063655 10.1016/j.drup.2022.100863

[18] Anderson, M. G. et al. Targeting multiple EGFR-expressing tumors with a highly potent tumor-selective antibody-drug conjugate. *Mol. Cancer Ther.* **19**(10), 2117–2125. 10.1158/1535-7163.MCT-20-0149 (2020). 32847977 10.1158/1535-7163.MCT-20-0149

[19] Gan, H. K. et al. Targeting of a conformationally exposed, tumor-specific epitope of EGFR as a strategy for cancer therapy. *Cancer Res.* **72**(12), 2924–2930. 10.1158/0008-5472.CAN-11-3898 (2012). 22659454 10.1158/0008-5472.CAN-11-3898

[20] Ho, E. C. H. et al. Characterization of monoclonal antibodies generated to the 287–302 amino acid loop of the human epidermal growth factor receptor. *Antibody Ther.* **2**(4), 88–98. 10.1093/abt/tbz011 (2019). 10.1093/abt/tbz011 PMC6947844 31934685

[21] Lorimer, I. A. et al. Immunotoxins that target an oncogenic mutant epidermal growth factor receptor expressed in human tumors. *Clin. Cancer Res.* **1**(8), 859–864 (1995). 9816055

[22] Ekstrand, A. J. et al. Amplified and rearranged epidermal growth factor receptor genes in human glioblastomas reveal deletions of sequences encoding portions of the N- and/or C-terminal tails. *Proc. Natl. Acad. Sci. U. S. A.* **89**(10), 4309–4313. 10.1073/pnas.89.10.4309 (1992). 1584765 10.1073/pnas.89.10.4309 PMC49071

[23] Nishikawa, R. et al. A mutant epidermal growth factor receptor common in human glioma confers enhanced tumorigenicity. *Proc. Natl. Acad. Sci. U. S. A.* **91**(16), 7727–7731. 10.1073/pnas.91.16.7727 (1994). 8052651 10.1073/pnas.91.16.7727 PMC44475

[24] Garrett, T. P. et al. Antibodies specifically targeting a locally misfolded region of tumor associated EGFR. *Proc. Natl. Acad. Sci. U. S. A.* **106**(13), 5082–5087. 10.1073/pnas.0811559106 (2009). 19289842 10.1073/pnas.0811559106 PMC2656555

[25] Ferguson, K. M. et al. EGF activates its receptor by removing interactions that autoinhibit ectodomain dimerization. *Mol. Cell* **11**(2), 507–517. 10.1016/s1097-2765(03)00047-9 (2003). 12620237 10.1016/s1097-2765(03)00047-9

[26] Lim, Y. et al. GC1118, an anti-EGFR antibody with a distinct binding epitope and superior inhibitory activity against high-affinity EGFR ligands. *Mol. Cancer Ther.* **15**(2), 251–263. 10.1158/1535-7163.MCT-15-0679 (2016). 26586721 10.1158/1535-7163.MCT-15-0679

[27] Walker, F. et al. CR1/CR2 interactions modulate the functions of the cell surface epidermal growth factor receptor. *J. Biol. Chem.* **279**(21), 22387–22398. 10.1074/jbc.M401244200 (2004). 15016810 10.1074/jbc.M401244200

[28] Ogiso, H. et al. Crystal structure of the complex of human epidermal growth factor and receptor extracellular domains. *Cell* **110**(6), 775–787. 10.1016/s0092-8674(02)00963-7 (2002). 12297050 10.1016/s0092-8674(02)00963-7

[29] Garrett, T. P. et al. Crystal structure of a truncated epidermal growth factor receptor extracellular domain bound to transforming growth factor alpha. *Cell* **110** (6), 763–773. 10.1016/s0092-8674(02)00940-6 (2002). 12297049 10.1016/s0092-8674(02)00940-6

[30] Bagchi, A. et al. Structural insights into the role and targeting of EGFRvIII. *Structure* **32**(9), 1367-1380 e6. 10.1016/j.str.2024.05.018 (2024). 38908376 10.1016/j.str.2024.05.018 PMC11380598

[31] Feng, J. et al. Feedback activation of EGFR/wild-type RAS signaling axis limits KRAS(G12D) inhibitor efficacy in KRAS(G12D)-mutated colorectal cancer. *Oncogene* **42**(20), 1620–1633. 10.1038/s41388-023-02676-9 (2023). 37020035 10.1038/s41388-023-02676-9 PMC10181928

[32] Young, A., Lou, D. & McCormick, F. Oncogenic and wild-type Ras play divergent roles in the regulation of mitogen-activated protein kinase signaling. *Cancer Discov.* **3**(1), 112–123. 10.1158/2159-8290.CD-12-0231 (2013). 23103856 10.1158/2159-8290.CD-12-0231

[33] Ho, E. C. H. et al. Antibody drug conjugates, targeting cancer-expressed EGFR, exhibit potent and specific antitumor activity. *Biomed. Pharmacother.* **157**, 114047. 10.1016/j.biopha.2022.114047 (2023). 36459711 10.1016/j.biopha.2022.114047 PMC9840435

[34] Otwinowski, Z. & Minor, W. Processing of X-ray diffraction data collected in oscillation mode. *Methods Enzymol.* **276**, 307–326. 10.1016/S0076-6879(97)76066-X (1997). 27754618 10.1016/S0076-6879(97)76066-X

[35] Long, F. et al. BALBES: a molecular-replacement pipeline. *Acta Crystallogr. D Biol. Crystallogr.* **64** (Pt 1), 125–132. 10.1107/S0907444907050172 (2008). 18094476 10.1107/S0907444907050172 PMC2394813

[36] Collaborative Computational Project, Number 4. The CCP4 suite: programs for protein crystallography. *Acta Crystallogr D Biol Crystallogr* **50**(Pt 5), 760–763. 10.1107/S0907444994003112 (1994). 15299374 10.1107/S0907444994003112

[37] Emsley, P. & Cowtan, K. Coot: model-building tools for molecular graphics. *Acta Crystallogr D Biol Crystallogr* **60**(Pt 12 Pt 1), 2126–2132. 10.1107/S0907444904019158 (2004). 15572765 10.1107/S0907444904019158

[38] Adams, P. D. et al. PHENIX: Building new software for automated crystallographic structure determination. *Acta Crystallogr. D Biol. Crystallogr.* **58**(Pt 11), 1948–1954. 10.1107/s0907444902016657 (2002). 12393927 10.1107/s0907444902016657

