# Supplementary material for: Targeting cancer expressed EGFR with a humanized monoclonal antibody
Source: Sci Rep. 2026 Mar 28;16:10814. doi: 10.1038/s41598-026-46245-y (PMC13040018; doi:10.1038/s41598-026-46245-y)

Targeting cancer-expressed EGFR with a humanized monoclonal antibody.

Tamara G. Fernandes Costa[1], Robert Sarnovsky[1], Jingyu Zhan[2], Carolyn A. Maslanka[2], Ifechukwu Obiorah[1], Di Xia[2], David FitzGerald[1], Antonella Antignani[1,*]

[1]Laboratory of Molecular Biology, Center for Cancer Research, National Cancer Institute, National Institutes of Health, Bethesda, MD 20892-2590, USA.
[2]Laboratory of Cell Biology, Center for Cancer Research, National Cancer Institute, National Institutes of Health, Bethesda, MD 20892-2590, USA.
*corresponding author

Supplementary Materials and Methods

Flow cytometry analysis.

Antibodies were incubated with suspended cells (2 $\times 10^5$ cells per well) in a 5ml polystyrene round bottom tube on ice for 1 h in FACS buffer consisting of PBS (KD Medical, MD, USA), 1% BSA (Sigma-Aldrich, MO, USA) and 0.1% sodium azide (Sigma-Aldrich, MO, USA). The humanized and cetuximab antibodies were detected with R-Phycoerythrin AffiniPure F(ab')$_2$ Fragment Goat Anti-Human IgG (H+L) while 40H3 was detected R-Phycoerythrin AffiniPure F(ab')$_2$ Fragment Goat Anti-mouse IgG (H+L) both from Jackson ImmunoResearch, ME, USA) at a 1:200 dilution for 30 min on ice. Each sample was then incubated with the viability dye eFluor R780 at a dilution of 1:700 (ThermoFisher Scientific, NY, USA) for 15 min on ice. Antibody binding was characterized using a BD FACSCantoII Flow Cytometer (BD Bioscience, San Jose, CA, USA) and the data were analyzed with FlowJo software (Tree Star, Inc., Ashland, OR USA).

To calculate the number of surface binding sites per cell, A10, 40H3 and cetuximab were each conjugated to R-Phycoerythrin using the R-Phycoerythrin Conjugation Lightning Kit following manufacturer's direction (Abcam, Cat ab102918). The labeled antibodies were incubated with cells (2.5 $\times 10^5$ cells per tube) on ice for 1 h in FACS buffer. Quantibrite beads (BD Bioscience. Cat# 340495) were used as reference for quantifying binding sites on cells. Antibody binding was characterized with the BD FACSCanto II Flow Cytometer (BD Bioscience, CA, USA), the data were analyzed with FlowJo software (Tree Star, Inc., Ashland, OR, USA) and binding site quantification was determined with Excel (Microsoft, USA) and GraphPad Prism Software, (La Jolla California USA).

ADC construction

Purified A10 antibody was conjugated to the tubulin-disrupting compound, MMAE, and conjugation reactions were performed under contract at NJBio, Princeton, NJ. Briefly, for conjugation via internal and reduced disulfides, the A10 antibody were subjected to mild reduction (4.5 molar excess of TCEP to antibody in PBS). Samples were desalted using Zeba columns (ThermoFisher Scientific, NY, USA) according to the manufacturer's instructions and then conjugated via maleimide groups on the linker payload entity. Specifically, MMAE, was linked to A10 via a cathepsin cleavable linker. The free drug payload was produced by NJBio and dissolved in DMSO to a final stock concentration of 1mM. hIgG1-MMAE was purchased from CellMosaic (Woburn, MA, USA)

Cell viability

5000 cells per well in a volume of 100 µl were plated in 96-well tissue culture plates. After 24 h, A10-MMAE, unconjugated MMAE or hIgG1-MMAE were added at the indicated concentrations. After 72 h, the medium was removed and viability was determined using the CellTiter -Glo luminescent assay kit (Promega, Madison, WI). This assay quantifies the amount of ATP present in metabolically active cells. ATP was detected as luminescence produced by the mono-oxygenation of luciferin which was catalyzed by the Ultra-Glo-luciferase. The luminescence of each well was determined, and the values were presented as a percentage relative to untreated cells (control).

Supplemental Figure Legend:

Supplementary Figure 1: **40H3 humanization design.** a) Amino acid sequence of the humanized variable heavy chains (VH1, VH2, VH3), light chains (VL1, VL2, VL3) and the two additional light chain variants D56A and E57G. The CDR regions are in blue and mutated amino acids for humanization are in red. b) Amino acidic sequence of the human IgG1Constant Heavy /Ig kappa Constant Light acceptor.

Supplementary Figure 2: Zoom-in view of the interactions between the EGFR peptide and Fab(A10). The Fab heavy and light chains are rendered as cartoon models with a semi-transparent surface and colored cyan and green, respectively. Side chains of key residues that interact with EGFR peptide are shown as stick models and labeled. The EGFR peptide is colored magenta, with side chains shown as sticks and residues numbered according to their positions in full-length EGFR.

Supplementary Figure 3: The oxidized EGFR peptide bound to Fab(A10) adopts a β-hairpin structure that closely matches the corresponding region in the full-length EGFR ectodomain. The Fab heavy chain and light chains are shown in purple and pink, respectively. The EGFR peptide is colored green and overlaid with the same loop region from published EGFR structures (1MOX, cyan; 1IVO, orange; 1NQL, blue; 4UV7, red).

Supplementary Figure 4: **A10 ADC characterization.** a) A10-MMAE structure and binding to cells. Increasing concentrations of A10-MMAE exhibited the same binding activity as the unmodified A10 antibody. b) MDA-MB-468 and A431 were treated in a dose dependent manner with A10-MMAE, the payload, MMAE or hIgG1-MMAE for 72 hours. c) A panel of EGFR-expressing tumor cells and the wild type EGFR normal fibroblast cell line, WI38, were treated for 72h with increasing concentrations of A10-MMAE or the payload, MMAE. The mean values were determined from at least three different experiments where each point in each experiment was derived from wells in triplicate. The error bars represent the standard deviation of the mean.

Supplementary Movie 1: EGFR conformations and activation. Morphing of conformational transition of EGFR ectodomain from monomeric, tethered state (PDB: 4UV7) to dimeric, untethered state (PDB: 3NJP).

Supplementary Movie 2: A10 binds to the full-length EGFR during the transition from tethered to untethered state. Upon ligand binding, the receptor undergoes a substantial domain rearrangement, transitioning from monomeric untethered to dimeric tethered form, exposing the epitope in the transition process which permits antibody A10 binding.

	806			A10		
EGFR peptide residue	Light Chain residue	Heavy Chain residue	CDRs	Light Chain residue	Heavy Chain residue	CDRs
A289 (N)	N30 (OD1)		L1			
E293 (OE1, OE2)	H50 (NE2)		L2	W32 (NE1)		L1
E295 (OE1, OE2)					K98 (NZ)	H3
E296 (OE2)					R53 (NE)	H3
D297(OD2)		Y51 (OH)	H2		G33 (N*)	H1
D297					R53 (N*)	H2
D297 (O*)					H35 (NE2)	H1
G298					G103 (N*)	H3
R300 (NH1, NH2)		D32 (OD2, OD1)	H1	Y92(O*, CE2)		L3
R300 (N*)		G100 (O*)	H3	L91(O*)		L3
K301 (NZ)	H2O216-Y91(O*)		L3		W52 (CD2)	H2
K301 (NZ)	H2O216-W96 (NE1)		L3			
C302 (O*)	N32 (ND2)		L1			
C302 (N*)	N32 (OD1)		L1			

Supplementary Table 1. Differences in interactions of the EGFR peptide between A10 and 806. (*) are the main chain atoms. CDR represent the Complementarity Determining Regions. Van der Waals' contacts are not listed

	**1MOX**	**1IVO**	**4UV7**	**1NQL**
A10-Peptide	1.7	0.9	0.7	1.32

Supplementary Table 2. Structural alignment (rmsd, Å) of the 287-302 peptide bound to A10 compared with to its counterpart in various EGFR structures.

SUPPLEMENTARY FIGURE 1

a)

```
Mouse 40H3 VH      QVQLKQSGPGLVQPSQSLSITCTVSGFSLTNYGIHWLRQSPGKGLEWLGMMWRGGGTDYN  60
Humanized VH1      QVTLKESGPVLVKPTETLTLTCTVSGFSLTNYGIHWIRQPPGKALEWLAMMWRGGGTDYN  60
Humanized VH2      QVTLKESGPVLVKPTETLTLTCTVSGFSLTNYGIHWIRQPPGKALEWLGMMWRGGGTDYN  60
Humanized VH3      QVTLKESGPVLVKPTETLTLTCTVSGFSLTNYGIHWLRQPPGKALEWLGMMWRGGGTDYN  60
                   ** **:*** **:*:::*::****************:** ***.****.***********

Mouse 40H3 VH      AAFISRLTITKDTSKSQVFFRMNNLQTNDTAIYYCARKGVGMGLGYWGQGTSVTVSS    117
Humanized VH1      AAFISRLTISKDTSKSQVVLTMTNMDPVDTATYYCARKGVGMGLGYWGQGTLVTVSS    117
Humanized VH2      AAFISRLTITKDTSKSQVVLTMTNMDPVDTATYYCARKGVGMGLGYWGQGTLVTVSS    117
Humanized VH3      AAFISRLTITKDTSKSQVVFTMTNMDPVDTATYYCARKGVGMGLGYWGQGTLVTVSS    117
                   *********:********.: *.*::  *** ******************** *****
```

```
Mouse 40H3 VL      DIQMTQSPASQSASLGESVTITCLASQTIGTWVAWYQQKPGRSPQLLIYGATNLADGVPS  60
Humanized VL1      DIQMTQSPSSVSASVGDRVTITCLASQTIGTWVAWYQQKPGKAPKLLIYGATNLADGVPS  60
Humanized VL1-DA   DIQMTQSPSSVSASVGDRVTITCLASQTIGTWVAWYQQKPGKAPKLLIYGATNLADAVPS  60
Humanized VL1-EG   DIQMTQSPSSVSASVGDRVTITCLASQTIGTWVAWYQQKPGKAPKLLIYGATNLAEGVPS  60
Humanized VL2      DIQMTQSPSSVSASVGDRVTITCLASQTIGTWVAWYQQKPGKSPQLLIYGATNLADGVPS  60
Humanized VL3      DIQMTQSPSSVSASVGDRVTITCLASQTIGTWVAWYQQKPGKSPQLLIYGATNLADGVPS  60
                   ********:* ***:*: ***********************::*:**********:.***

Mouse 40H3 VL      RFSGSGSGTKFSFKISSLQAEDFVSYYCQQLYSNPYTFGGGTKLEIK     107
Humanized VL1      RFSGSGSGTDFTLTISSLQPEDFATYYCQQLYSNPYTFGGGTKLEIK     107
Humanized VL1-DA   RFSGSGSGTDFTLTISSLQPEDFATYYCQQLYSNPYTFGGGTKLEIK     107
Humanized VL1-EG   RFSGSGSGTDFTLTISSLQPEDFATYYCQQLYSNPYTFGGGTKLEIK     107
Humanized VL2      RFSGSGSGTDFTLTISSLQPEDFATYYCQQLYSNPYTFGGGTKLEIK     107
Humanized VL3      RFSGSGSGTKFTLTISSLQPEDFATYYCQQLYSNPYTFGGGTKLEIK     107
                   *********.*::.***** ***.:***********************
```

b)

hIgG1CH

ASTKGPSVFPLAPSSKSTSGGTAALGCLVKDYFPEPVTVSWNSGALTSGVHTFPAVLQSSGLYSLSS
VVTVPSSSLGTQTYICNVNHKPSNTKVDKKVEPKSCDKTHTCPPCPAPELLGGPSVFLFPPKPKDTL
MISRTPEVTCVVVDVSHEDPEVKFNWYVDGVEVHNAKTKPREEQYNSTYRVVSVLTVLHQDWLNG
KEYKCKVSNKALPAPIEKTISKAKGQPREPQVYTLPPSRDELTKNQVSLTCLVKGFYPSDIAVEWESN
GQPENNYKTTPPVLDSDGSFFLYSKLTVDKSRWQQGNVFSCSVMHEALHNHYTQKSLSLSPGK

hIgκCL

RTVAAPSVFIFPPSDEQLKSGTASVVCLLNNFYPREAKVQWKVDNALQSGNSQESVTEQDSKDSTY
SLSSTLTLSKADYEKHKVYACEVTHQGLSSPVTKSFNRGEC

SUPPLEMENTARY FIGURE 2

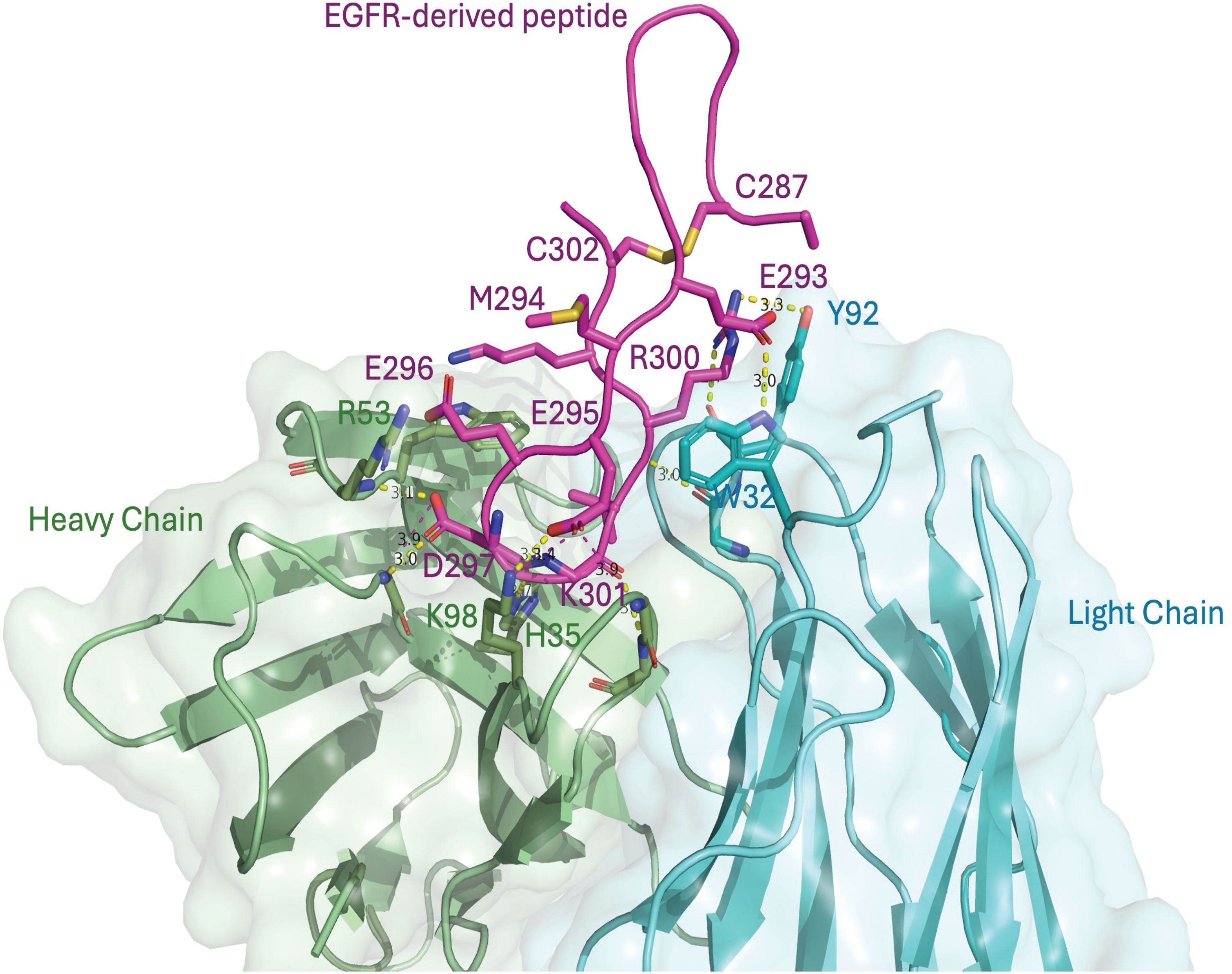

SUPPLEMENTARY FIGURE 3

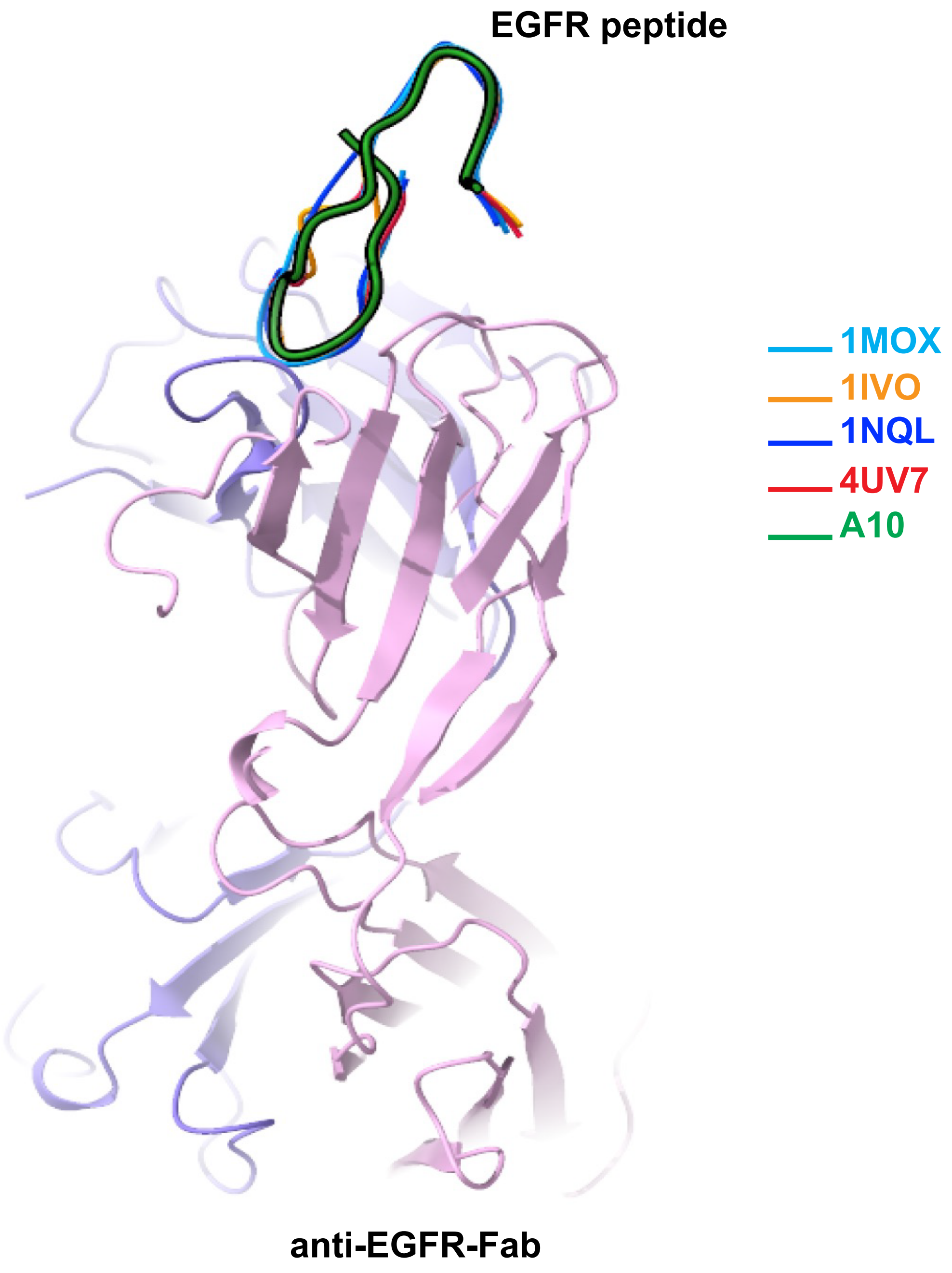

SUPPLEMENTARY FIGURE 4

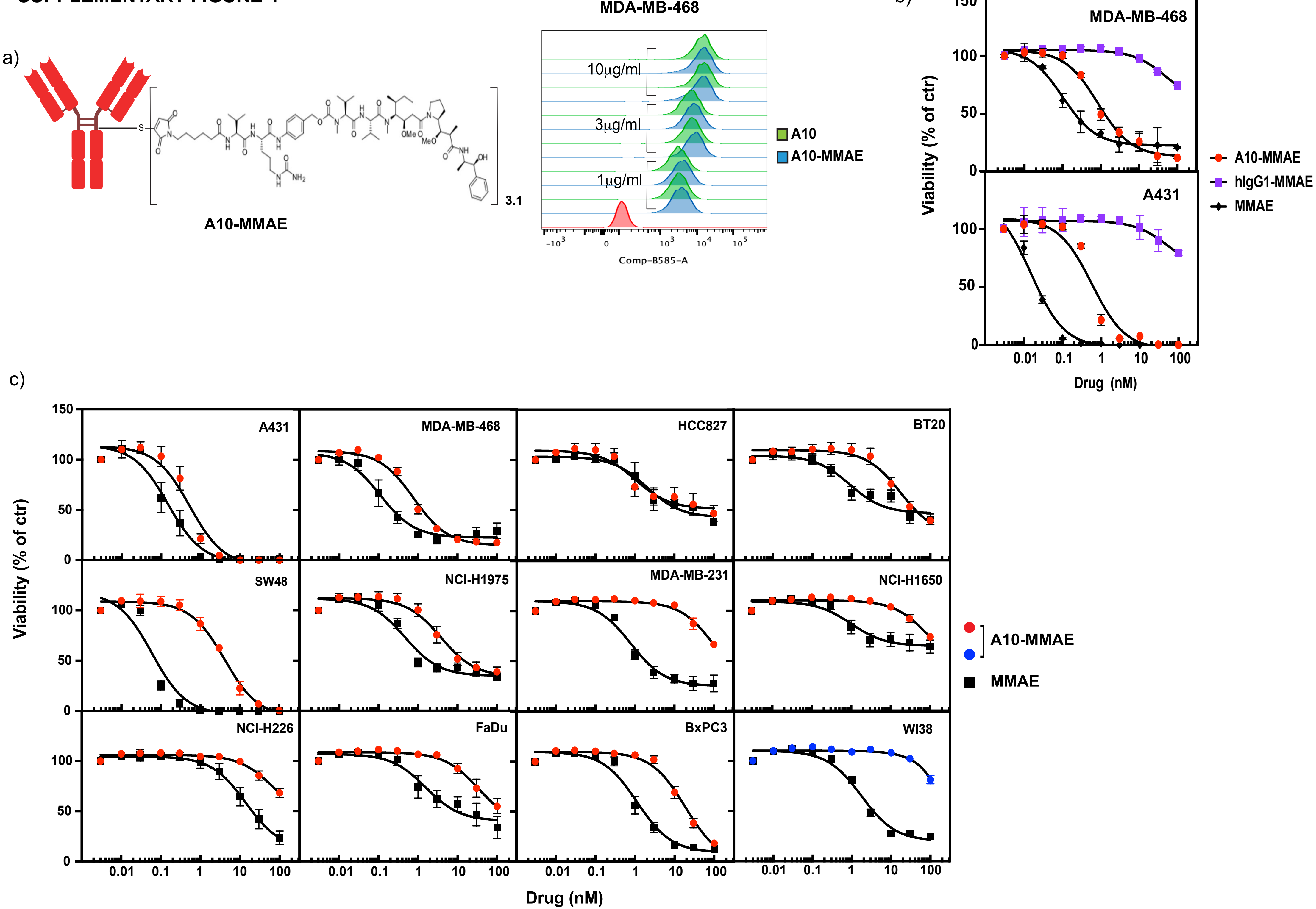

Supplement: Supplementary file 3 — Supplementary Material 3 [file 41598_2026_46245_MOESM3_ESM.pdf]
